# Now or Later? The Role of Neoadjuvant Treatment in Advanced Endometrial Cancer: A Systematic Review

**DOI:** 10.3390/healthcare12232404

**Published:** 2024-11-29

**Authors:** Carlo Ronsini, Irene Iavarone, Alessandro Carotenuto, Antonio Raffone, Giada Andreoli, Stefania Napolitano, Pasquale De Franciscis, Domenico Ambrosio, Luigi Cobellis

**Affiliations:** 1 Department of Woman, Child and General and Specialized Surgery, University of Campania “Luigi Vanvitelli”, 80138 Naples, Italy; ireneiavarone2@gmail.com (I.I.); alessandro-grande@hotmail.it (A.C.); antonio.raffone@unicampania.it (A.R.); andreoli.giada@gmail.com (G.A.); pasquale.defranciscis@unicampania.it (P.D.F.); domenico.ambrosio@unicampania.it (D.A.); luigi.cobellis@unicampania.it (L.C.); 2 Medical Oncology Unit, Department of Precision Medicine, University of Campania “Luigi Vanvitelli”, 80138 Naples, Italy; stefania.napolitano@unicampania.it

**Keywords:** endometrial cancer, upfront surgery, chemotherapy, radiotherapy, neoadjuvant

## Abstract

**Background:** Endometrial cancer (EC) is, nowadays, the most frequent gynecological malignancy worldwide. The main treatment approach for EC is surgery, especially for early-stage tumors. For advanced EC, chemotherapy (CT) with carboplatin and paclitaxel is the standard treatment, especially for women with metastatic or recurrent disease. The present systematic review aimed to establish whether neoadjuvant treatment regimens with CT and/or radiotherapy (RT) lead to better survival outcomes compared to upfront surgery in advanced EC. **Methods:** Following the Preferred Reporting Items for Systematic Reviews and Meta-Analyses (PRISMA) statement, through the string “((“Endometrial Neoplasms”[Mesh]) AND “Hysterectomy”[Mesh]) AND “Radiotherapy”[Mesh] AND Chemotherapy”, the selection of articles was made. A quality assessment was conducted using the Newcastle–Ottawa Scale (NOS). The studies included patients with EC with survival and recurrence outcomes—patients treated with upfront surgery or neoadjuvant CT ± External Beam Radiation Therapy (EBRT) or CT ± Brachytherapy (BT). **Results:** According to the selected evidence in the scientific literature, the 5-year DFS was 21.3% for upfront surgery and ranged from 42 to 73% for neoadjuvant chemotherapy. Also, the 5-year OS was 6.2 to 49.7% with upfront surgery and 15.5 to 100% for neoadjuvant schemes. None of the studies dedicated to surgery reported the 5-year Recurrence Rate (RR), while in the neoadjuvant treatments, it ranged from 27 to 64.7%. **Conclusions:** The literature’s paucity of data makes it difficult to compare neoadjuvant therapy regimens with upfront surgery in advanced endometrial carcinoma. Nevertheless, the current data show more encouraging results for the neoadjuvant treatment group.

## 1. Introduction

Endometrial cancer (EC) is now the most frequent gynecological malignancy in the world [1].

Despite the high incidence of this carcinoma, most diagnoses are confined to the uterus and have an excellent survival profile [2]. Nevertheless, when the disease presents in an advanced and metastatic form, whether lymph node or peritoneal metastases, the prognostic impact of endometrial carcinoma changes dramatically [3]. International guidelines suggest cytoreductive surgery followed by adjuvant chemotherapy and radiotherapy treatments [4,5]. This approach is not universally recognized, and some schools of thought prefer to subject patients with metastatic endometrial carcinoma to cycles of neoadjuvant treatment before cytoreductive surgery [5,6,7,8,9,10]. Recently, much attention has been paid to the use of immunotherapy in EC [11,12]. The induction of this new technology offers new therapeutic horizons. However, it does not currently find use in the management of advanced carcinomas, but forces us to reconsider what is currently known about the management of endometrial carcinoma. In fact, the use of new therapeutic schemes would not make sense if we did not first clarify which should be considered the gold standard today. To date, there is no unanimity in the literature on the advantages of surgery or neoadjuvant chemotherapy. Moreover, there is no standardized chemotherapy scheme for the management of these patients. Our review aims to explore the advantages and disadvantages of these two strategies and to understand which offers better oncological outcomes.

## 2. Materials and Methods

The materials and methods for this study were specified a priori based on the recommendations in the Preferred Reporting Items for Systematic Reviews and Meta-Analyses (PRISMA) statement [13].

### 2.1. Search Method

We conducted a systematic search regarding the treatment modalities of advanced endometrial carcinomas in PubMed, Scopus, and EMBASE Datasets in July 2024. All papers published since the oldest one were included. This covered 23 years. No restriction of the country was performed. Only English fully available studies were considered. Search inputs were “((“Endometrial Neoplasms”[Mesh]) AND “Hysterectomy”[Mesh]) AND “Radiotherapy”[Mesh] AND Chemotherapy[Mesh]”.

### 2.2. Study Selection

**Figure 1:**
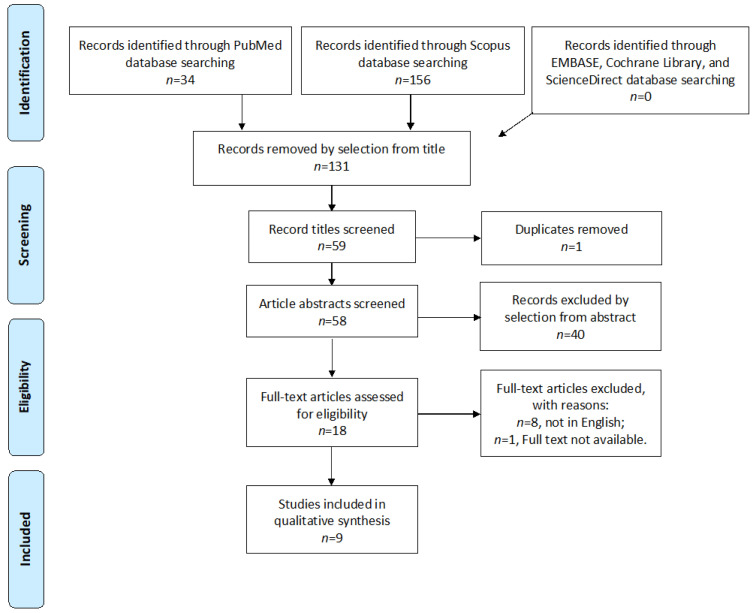
Preferred Reporting Items for Systematic Reviews and Meta-Analyses (PRISMA) Flow-Chart.

Study selection was performed independently by I.I. and C.A. In case of discrepancy, C.R. decided to include or exclude studies. Inclusion criteria were as follows: (1) studies that included patients with EC—patients treated with upfront surgery or neoadjuvant CT ± External Beam Radiation Therapy (EBRT) or CT ± brachytherapy (BT); (2) studies that reported at least one outcome of interest—5-year disease-free survival (DFS) (%), 5-year overall survival (OS) (%), 5-year Recurrence Rate (RR) (%); (3) peer-reviewed articles, published originally; (4) patients with stage III and IV disease that was histologically diagnosed. Non-original studies, preclinical trials, animal trials, abstract-only publications, and articles in a language other than English were excluded. If possible, contact was attempted with the authors of studies that were only published as congress abstracts via email and they were asked to provide their data. The studies selected and all reasons for exclusion are mentioned in the Preferred Reporting Items for Systematic Reviews and Meta-Analyses (PRISMA) Flow-Chart (Figure 1). All included studies were assessed regarding potential Conflicts of Interest.

### 2.3. Quality Assessment

The Newcastle–Ottawa Scale (NOS) [14] was used to assess the quality of the included studies. This assessment scale uses three broad factors (selection, comparability, and exposure), with scores ranging from 0 (lowest quality) to 8 (best quality) [14]. The NOS is reported in the Appendix A. AF and GA extracted the data for all relevant series and case reports, as well as data on tumor characteristics (size, stage, histological subtype, grading), surgical approach, residual tumor, chemotherapy and/or radiation schemes, morbidity, and oncological issues such as recurrences, deaths, RR, and CRR to chemotherapy treatment (CR); however, this activity was hindered by different criteria across papers and a diffused lack of information.

## 3. Results

### 3.1. Characteristics of Studies

After the database search, a total of 190 articles matched the search criteria. After removing records with no full text, duplicates, and wrong study designs (e.g., reviews), 59 were suitable for eligibility. Nine matched the inclusion criteria and were included in the systematic review. All of them were non-comparative, single-armed studies evaluating the survival outcomes of upfront surgery or neoadjuvant CT ± RT (Figure 1). The countries where the studies were conducted, the publication year range, the studies’ design, number of participants, FIGO Stage, type of treatment, and follow-up (FU) time are summarized in Table 1. The quality of all studies was assessed using the Newcastle–Ottawa Scale (NOS) (Table A1 in Appendix A). Overall, the publication years ranged from 2000 to 2023. In total, 878 patients—275 treated with upfront surgery and 603 with neoadjuvant treatment—with advanced-stage EC were enrolled and analyzed and the FU period ranged from 12.0 to 60.0 months, on average.

### 3.2. Outcomes

The systematic review included 878 patients—275 treated with upfront surgery and 603 with neoadjuvant treatment. Tumor staging was surgical in all cases. We divided the studies with surgery upfront from the studies with neo-adjuvant treatment. Regarding the survival outcomes, only four studies revealed the DFS of their cohort [15,16,18,19], whereas all the articles examined the OS. In the upfront surgery group, the 5-year DFS was only reported by Bristow et al. [15], with a value of 23.1%. In the neoadjuvant treatment group, the values ranged from 42.0% to 73.0%.

Regarding the OS, the surgery group showed survival values between 6.2% and 49.7%; conversely, the neoadjuvant treatment group showed values between 15.5% and 100%. No study on surgery reported data on the five-year risk of recurrence. The reported values ranged from 27.0% to 64.6% in the neoadjuvant treatment group. Unfortunately, no work under review provided data on the toxicity profile and the risk of complications. Table 2 summarizes the survival and recurrence outcomes.

### 3.3. Type of Surgery

In Bristow et al.’s study, the DFS was 23.1%, whereas the OS was 6.2% after cytoreductive surgery [15]. Entire hysterectomy was performed in 61 of the 65 women: besides hysterectomy, 6 had radical hysterectomy and 4 underwent additional resection of the rectosigmoid region. Three women underwent omentectomy and one had bilateral salpingo-oophorectomy because the tumor was unresectable [15]. In Alagkiozidis’ analysis, only the OS was assessed and it was 26% [17]. In particular, of 168 patients, 7 underwent radical hysterectomy. Pelvic and/or para-aortic lymph node dissection was applied in 114. Ninety-eight women underwent omentectomy. Also, 20 bowel resections, 1 splenectomy, 1 resection of liver segment, and 1 diaphragmatic resection were performed. In Zhu et al.’s cohort, the 5-year OS was 49.7% [18]. Due to the heterogeneity of the data and tumor stage, no additional information about cytoreduction was provided [18].

### 3.4. Type of Neoadjuvant Treatment

In Vargo et al.’s study, despite the extensive presentations of EC and CT administration, data supporting the use of neoadjuvant EBRT ± CT regarding pathologic response, tumor control, and survival were scarce [16]. Only one local failure was registered, and the 3-year local control rate was 96% [16]. In particular, the patients were administered with EBRT (45–50.4 Gy in 25 to 28 fractions), image-based HDR BT (5–5.5 Gy in 3 to 4 fractions), and carboplatin + paclitaxel (86% of the patients), gemcitabine + Taxotere (7% of the patients), bevacizumab (7% of the patients) [17]. In Conway et al.’s study, the patients underwent neoadjuvant CT or RT, and the 5-year DFS and OS were 42% and 70%, respectively [19]. The RR was 58% at 5 years [19]. Iheagwara et al. analyzed the outcomes of neoadjuvant CT: the DFS was 52.5%, the OS was 63.7%, and the RR was 47.5% [22]. In particular, the patients were administered with EBRT (45–50.4 Gy in 25 to 28 fractions) and high-dose-rate BT with a median total dose of 20 Gy (range 15–27.5) in 3 to 5 fractions [20]. CT consisted of platinum [20]. Rauh et al. analyzed the outcomes of neoadjuvant CT as well, and the OS was 31.6%, whereas the RR was 64.6% [21]. In Sakai et al.’s study, the patients were administered with neoadjuvant EBRT or BT [22]. The 5-year OS was 15.5% [22]. In Mevius et al.’s study, the patients were administered with neoadjuvant platinum CT and a 33.2% OS was reported [23].

## 4. Discussion

### 4.1. Summary of Main Results

The literature lacks comparative studies, making it difficult to identify an ideal treatment. This evidence is further marred by the lack of standardization of neoadjuvant treatment schemes, which makes scientific evidence fragmentary and episodic. Nevertheless, in the reported series, the survival is better with the neoadjuvant approaches than with upfront surgery. This could be justified by the appropriateness of the surgical procedure, which, in the face of advanced pathology, may not achieve complete cytoreduction. Moreover, the degree of complexity of this surgery may reflect debilitatingly on the patient, slowing the healing time and thus the adherence to systemic treatment protocols.

### 4.2. Results in the Context of Published Literature

Due to the low incidence of advanced-stage EC, there is no consensus on which would be the optimal treatment strategy: upfront surgery followed by chemotherapy and radiotherapy, or neoadjuvant therapy followed by debulking. The 2014 National Comprehensive Cancer Network Clinical Practice Guidelines recommend neoadjuvant high-dose-rate (HDR) BT with dose fractionation [24,25]. In contrast, the guidelines of the European Society of Gynaecological Oncology (ESGO) recommend cytoreduction as the first approach to the disease, when the surgeon envisages a complete resection [26]. This is similar to the current consensus in the treatment of advanced ovarian cancer [27]. The rationale behind such a strategy may be related to the macroscopic reduction in the pathology by facilitating the penetration of chemotherapy and, thus, its efficacy [28]. Moreover, it should be noted that none of the studies reviewed included immunotherapy schemes. The recent inclusion of Dostarlimab in treating advanced disease has shown a significant prognostic improvement [12,20,21,22]. Any consideration of the best therapeutic approach in advanced endometrial carcinoma should include this option.

### 4.3. Implications for Practice and Future Research

This shortcoming underlines how the treatment of advanced endometrial carcinomas still represents a gray area of clinical practice, where, in light of the low incidence of this presentation, there is a need to achieve therapeutic standards supported by scientific evidence. In addition, increasing the awareness of the molecular profiles of endometrial carcinoma will lead to a better estimation of their prognostic values [29]. At present, since advanced carcinoma is treated on the basis of its clinical manifestation independently of these other parameters, more personalization on the basis of the intrinsic characteristics of the aggressiveness of the various tumors will be necessary for the future. Only by expanding this knowledge will it be possible to supplement current treatment schemes with immunotherapy and hormone therapy. Finally, the design of comparative clinical trials will be the only useful step in determining the best approach for this disease. Unfortunately, the rarity of the presentation and the low prognostic expectations make the design of such research protocols difficult. Moreover, further perspectives may focus on the different options to adopt when there is no visible disease after neoadjuvant CT.

### 4.4. Strength and Limitations

The main limitations of the present study are the heterogeneity of data and the retrospective design of the selected studies [22,23]. In particular, it was impossible to adequately assess the impact of CT on survival outcomes [20,21,30]. Likewise, the brevity of the follow-up in most of the studies made true contextualization of the data difficult. However, this is the first analysis comparing upfront surgery with using neoadjuvant therapy in the advanced stage and covered all existing literature on the subject [19,27,28,29,30,31,32,33,34]. Even though we are aware of the differences in the surgical treatments across the years, our analysis includes all articles in the scientific literature. One limitation is the lack of standardization in the chemotherapy schemes. Unfortunately, this is unavoidable in view of the great variety of approaches to this disease in its advanced stages. Furthermore, within the same approach category, the various studies are difficult to compare due to the wide time span of the publications. This exposes methodological biases related to the advancement of medical technology and pharmacological knowledge. However, the more recent studies did not report significantly higher outcomes than the older studies. Our review offers a systematic overview of the data in the literature regarding the treatment of advanced endometrial carcinoma. Although incomplete, all the data in the literature have been included in the review. However, the review is a starting point to deepen this evidence through standardization, comparison, and prospective data analysis. Obviously, the absence of studies comparing upfront surgery with neoadjuvant therapy makes the results weak. This is the reason why we highlight the importance of performing further prospective studies, and also to analyze the impact on surgical outcomes.

Finally, none of the studies consider the molecular classification of treated carcinomas. Recent research on the prognostic impact of molecular classification in EC suggests that they may also have a different prognostic impact in advanced forms of carcinoma [35,36]. Moreover, forms with mismatch repair deficiency (MMRp) currently benefit from immunotherapeutic treatments [37]. Therefore, a sample stratification based on its molecular characteristics is desirable to understand the best approach.

## 5. Conclusions

The literature’s paucity of data makes it difficult to compare neoadjuvant therapy regimens with frontal surgery in advanced endometrial carcinoma. However, current data show more encouraging results for the neoadjuvant treatment group. However, this evidence is marred by the almost total lack of standardization of treatment schemes. On the other hand, the systematic nature of the review requires that everything that has been published is taken into consideration. The adopted methodology is itself part of the study design, and this makes our study the most updated review on this topic. Further studies involving standardization of neoadjuvant treatments, integration with immunotherapy protocols, and comparative designs will be necessary to resolve this dilemma.

## Figures and Tables

**Table 1:** Characteristics of included studies.

Author, Year of Publication	Country	Period of Enrollment	Study Design	FIGO Stage	Endometrioid Cancer (%)	RT 0 (%)	No. of Participants	Treatment	Median FU Period (Months)
Bristow 2000 [15]	USA	1990–1998	Retrospective Monocenter Cohort	IVB	33.8	55.4	65	Upfront Surgery	14.7
Vargo 2014 [16]	USA	1999–2014	Retrospective Monocenter Cohort	IIIB–IV	83	NR	36	Neoadjuvant EBRT ± CT	20
Alagkiozidis 2015 [17]	USA	1984–2009	Retrospective Monocenter Cohort	III–IV	60	64	168	Upfront Surgery	18
Zhu 2016 [18]	China	2006–2013	Retrospective Monocenter Cohort	III–IV	NR	NR	42	Upfront Surgery	49.2
Conway 2019 [19]	Canada	2000–2018	Retrospective Monocenter Cohort	II–IVA	59	NR	59	Neoadjuvant CT/RT	26
Iheagwara 2019 [20]	USA	2008–2018	Retrospective Monocenter Cohort	II–IV	NR	94	34	Neoadjuvant CT	60
Rauh 2020 [21]	USA	2000–2015	Retrospective Multicenter Cohort	III–IV	38.6	31	96	Neoadjuvant CT	24.4
Sakai 2022 [22]	Japan	2004–2011	Retrospective Multicenter Cohort	I–IV	NR	73.5	177	Neoadjuvant EBRT/BT	N/A
Mevius 2023 [23]	Germany	2010–2020	Retrospective Multicenter Cohort	III–IV	NR	NR	201	Neoadjuvant CT	12

FIGO: International Federation of Gynecology and Obstetrics; FU: follow-up; EBRT: External Beam Radiation Therapy; BT: brachytherapy; CT: chemotherapy. RT: residual tumor. NR: not reported.

**Table 2:** Outcomes.

Author, Year of Publication	Treatment	5-Year PFS (%)	5-Year OS (%)	5-Year RR (%)	National Cancer Institute Common Toxicity Criteria for Adverse Events ≥ 3 (%)
Bristow 2000 [15]	Upfront surgery	23.1	6.2	N/A	N/A
Vargo 2014 [16]	Neoadjuvant EBRT ± CT	73	100	27	9
Alagkiozidis 2015 [17]	Upfront surgery	N/A	26	N/A	N/A
Zhu 2016 [18]	Upfront surgery	N/A	49.7	N/A	N/A
Conway 2019 [19]	Neoadjuvant CT/RT	42	70	58	19
Iheagwara 2019 [20]	Neoadjuvant CT	52.5	63.7	47.5	N/A
Rauh 2020 [21]	Neoadjuvant CT	N/A	31.6	64.6	N/A
Sakai 2022 [22]	Neoadjuvant EBRT/BT	N/A	15.5	N/A	22
Mevius 2023 [23]	Neoadjuvant CT	N/A	33.2	N/A	N/A

PFS: progression-free survival; OS: overall survival; RR: Recurrence Rate.

## Data Availability

Not applicable.

## Appendix A

**Table A1:** Newcastle–Ottawa Scale.

Author, Year of Publication	Country	Selection	Comparability	Exposure	Total
Bristow 2000 [15]	USA	3	2	3	8
Vargo 2014 [18]	USA	3	2	3	8
Alagkiozidis 2015 [19]	USA	3	2	2	7
Zhu 2016 [20]	China	3	2	2	7
Conway 2019 [21]	Canada	3	2	3	8
Iheagwara 2019 [22]	USA	3	2	3	8
Rauh 2020 [23]	USA	3	2	2	7
Sakai 2022 [24]	Japan	2	2	2	6
Mevius 2023 [25]	Germany	3	2	2	7
